# The Role of Obesity and Diabetes in Dementia

**DOI:** 10.3390/ijms23169267

**Published:** 2022-08-17

**Authors:** Ashley Selman, Scott Burns, Arubala P. Reddy, John Culberson, P. Hemachandra Reddy

**Affiliations:** 1 Department of Internal Medicine, Texas Tech University Health Sciences Center, Lubbock, TX 79430, USA; 2 Nutritional Sciences Department, College Human Sciences, Texas Tech University, Lubbock, TX 79409, USA; 3 Department of Family Medicine, Texas Tech University Health Sciences Center, Lubbock, TX 79430, USA; 4 Department of Pharmacology and Neuroscience, Texas Tech University Health Sciences Center, Lubbock, TX 79430, USA; 5 Department of Neurology, Texas Tech University Health Sciences Center, Lubbock, TX 79430, USA; 6 Department of Public Health, Graduate School of Biomedical Sciences, Texas Tech University Health Sciences Center, Lubbock, TX 79430, USA; 7 Department of Speech, Language, and Hearing Sciences, Texas Tech University Health Sciences Center, Lubbock, TX 79430, USA

**Keywords:** Western diet, type 2 diabetes, dementia, obesity, depression, Mediterranean diet

## Abstract

Chronic conditions such as obesity, diabetes, and dementia are increasing in the United States (US) population. Knowledge of these chronic conditions, preventative measures, and proper management tactics is important and critical to preventing disease. The overlap between obesity, diabetes, and dementia is becoming further elucidated. These conditions share a similar origin through the components of increasing age, gender, genetic and epigenetic predispositions, depression, and a high-fat Western diet (WD) that all contribute to the inflammatory state associated with the development of obesity, diabetes, and dementia. This inflammatory state leads to the dysregulation of food intake and insulin resistance. Obesity is often the cornerstone that leads to the development of diabetes and, subsequently, in the case of type 2 diabetes mellitus (T2DM), progression to “type 3 diabetes mellitus (T3DM)”. Obesity and depression are closely associated with diabetes. However, dementia can be avoided with lifestyle modifications, by switching to a plant-based diet (e.g., a Mediterranean diet (MD)), and increasing physical activity. Diet and exercise are not the only treatment options. There are several surgical and pharmacological interventions available for prevention. Current and future research within each of these fields is warranted and offers the chance for new treatment options and a better understanding of the pathogenesis of each condition.

## 1. Introduction

**Figure 1:**
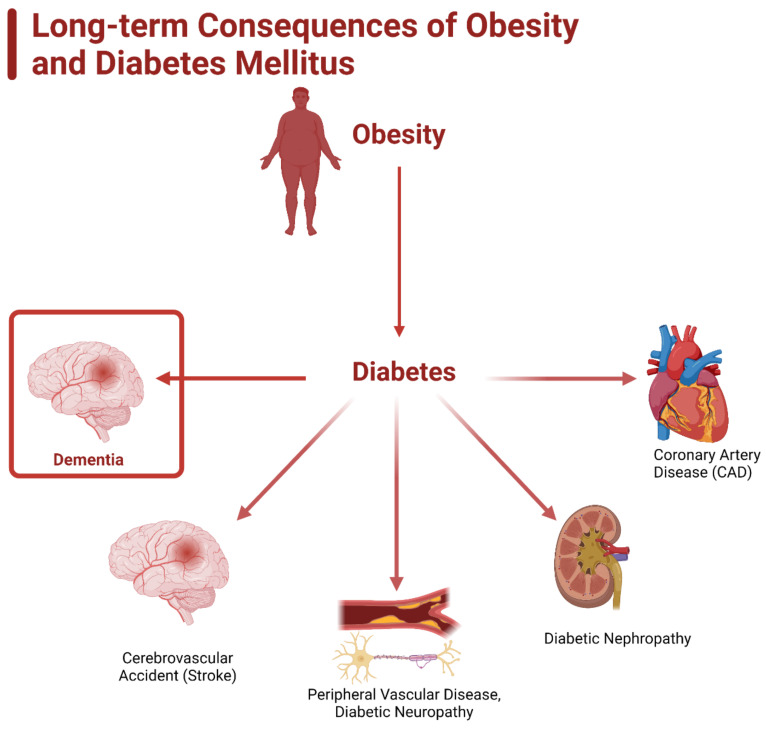
Long-term consequences of obesity and diabetes mellitus. Obesity is heavily linked as a causative agent of diabetes mellitus, especially type 2. Diabetes leads to the development of coronary artery disease (CAD), diabetic nephropathy, peripheral vascular disease, diabetic neuropathy, cardiovascular accident (stroke), and dementia.

Chronic diseases have become a serious problem in modern society and two of the most prominent conditions, diabetes mellitus and dementia, are closely linked to obesity. The Centers for Disease Control and Prevention (CDC) deems the steady rise in obesity within the United States (US) over the past few decades as an epidemic (Table 1) [1]. In 2017, 4 million people died due to obesity and obesity-related complications, with 41.9% of the US population being deemed obese [2]. Current research links the obesity epidemic to multiple detrimental chronic conditions (e.g., diabetes, heart disease, dementia) that have lowered the quality and length of life for countless individuals (Figure 1) [3]. As of 2020, 55 million people have been diagnosed with dementia and by 2050 this number is expected to rise to 139 million, likely due to the steady rise in obesity prevalence [3,4,5]. This article examines the factors surrounding the development of obesity and the role that obesity plays in perpetuating the prevalence of diabetes and dementia.

The rise in the incidence of obesity and diabetes leads to a corresponding increase in the risk of developing dementia. The table above was constructed utilizing data from the Centers for Disease Control and Prevention (CDC) with these parameters: (1) adults aged 20 years and older, (2) obesity prevalence calculated based upon only adults classified as having “obesity,” (3) diabetes mellitus prevalence calculated using data encompassing type 1 diabetes mellitus (T1DM) and type 2 diabetes mellitus (T2DM) as well as physician- and non-physician-diagnosed diabetes [5,6].

**Figure 2:**
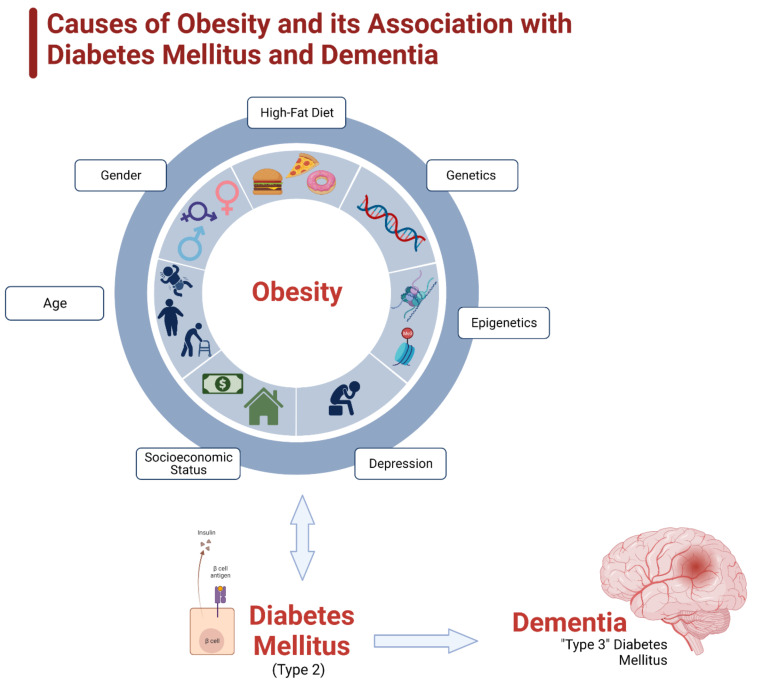
Causes of obesity and its association with diabetes mellitus and dementia. Obesity is a multifactorial condition, determined by not just an imbalance of caloric intake and usage, but also by several other factors. The main determining factors are age, gender, diet, genetic predisposition, epigenetic impact, depression, and socioeconomic status.

Obesity is defined as an “abnormal or excessive fat accumulation that presents a risk to health” and a body mass index (BMI) over 30. The term “overweight” is defined similarly, but with a BMI over 25. Obesity is most frequently caused by consuming an excess number of calories than is required for basal metabolic activities and physical exertion. However, all people are not created equal in this epidemic [2]. Some populations are disproportionately affected due to an interplay of several factors, such as age, gender, genetic and epigenetic factors, and the social determinants of health [1]. Many of these elements work concurrently to cause obesity, which in turn further bolsters the incidence of diabetes and dementia (Figure 2).

Extensive research cites several hypotheses linking obesity with the chronic conditions of diabetes. The most prominent and current of these hypotheses are the: (1) inflammation hypothesis, (2) lipid overflow hypothesis, and (3) adipokine hypothesis [6]. These hypotheses all center around the concept of increased insulin resistance, cytokines and other inflammatory mediators, lipid storage, and how obesity stimulates diabetes at the molecular level [7,8,9,10]. These cellular pathologies translate into the classical clinical presentation of hyperglycemia, which leads to polyuria, polydipsia, nocturia, and blurred vision [11]. Chronically elevated blood glucose levels lead to vascular damage, which causes further complications, such as coronary artery disease (CAD), peripheral neuropathy, stroke, and chronic kidney disease (Figure 1).

**Figure 3:**
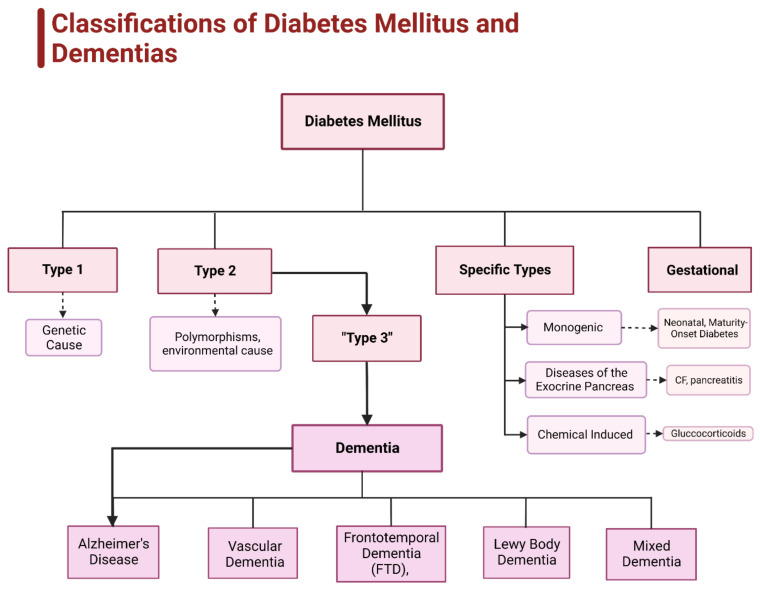
Classifications of diabetes mellitus and dementias. Figure illustrates the four types of diabetes mellitus and the relationship between type 2 diabetes mellitus (T2DM) and dementia, specifically Alzheimer’s disease (AD). T2DM has a close link to AD, leading to an umbrella type of diabetes known as “type 3 diabetes mellitus” (T3DM).

Diabetes can be subdivided into four categories: type 1, type 2, gestational, and specific cause-based types [12] (Figure 3). Type 1 diabetes is classified as an autoimmune disorder in which the body destroys insulin-producing pancreatic beta cells leading to an insulin deficiency, which also includes latent autoimmune diabetes in adulthood. Type 2 diabetes is the most common and is due to a combination of insulin resistance and a failure of pancreatic beta cells to secrete insulin [12,13]. “Type 3 diabetes” is a subset of type 2 and has been dubbed “brain diabetes” due to the strong correlation between type 2 diabetes and Alzheimer’s disease [13,14,15,16]. Gestational diabetes develops in pregnant women with no previous signs of diabetes who develop elevated glucose levels during the second or third trimester that typically resolves after giving birth. There are other types of diabetes linked to specific causes such as: (1) monogenic diabetes syndromes (e.g., neonatal diabetes, maturity-onset diabetes), (2) exocrine pancreas affecting disorders (e.g., cystic fibrosis, pancreatitis), and (3) chemical-induced diabetes (e.g., glucocorticoids) [11,12].

The chain reaction set off by obesity can be seen in the brain as dementia and other mental disorders like AD. Dementia is the overarching term to describe a group of neurological conditions that lead to a progressive decline in cognitive function and impairs the ability to reason and think, manage emotions, and can lead to a shift in personality. There are five common types of dementia: (1) AD, (2) frontotemporal dementia (FTD), (3) Lewy body dementia, (4) vascular dementia, and (5) mixed dementia [17] (Figure 3). Those suffering from type 2 diabetes have a 19% higher chance of specifically developing the vascular dementia form [17]. Obesity’s strong link to type 2 diabetes is what connects it to a decreased mental state. Type 2 diabetes increases the risk of developing a cognitive disorder by 60%, with a correlation so strong it has become another type of diabetes: type 3 [13,15,16,17].

This article investigates the role that obesity plays in perpetuating the prevalence of diabetes and dementia and the factors that contribute to the development of obesity in the population. In line with this goal, the following are discussed: (1) specific factors that affect the obesity rate in populations, including age, gender, genetics and epigenetics, nutrition, and the pertinence of socioeconomic factors, (2) the relationship between obesity, diabetes, and dementia, with a particular examination of the role of inflammation, (3) the impact of diabetes on dementia, (4) the current status of research on obesity, diabetes, and dementia, (5) research directions in the area of obesity and its role in diabetes and dementia, and (6) the current therapeutics (e.g., medications, diet, exercise) for diabetes and dementia.

## 2. Factors Affecting the Development of Obesity

### 2.1. Age and Obesity

Age and obesity have a convoluted and cyclic relationship. Individuals are more prone to experience an increase in central obesity as they age due to the decline in metabolic function [18]. Centralized obesity is a major contributor to key agents of oxidative stress-induced insulin resistance and metabolic syndrome [18]. The surgical removal of this visceral fat in mice counteracted the insulin resistance and occurrence of metabolic syndrome [18]. Moreover, obese individuals also have higher levels of inflammatory cytokines, which are shown to accelerate the aging process, creating a perpetually damaging cycle [19]. This inflammatory environment is created in part by the cytokines: tumor necrosis factor alpha (TNF-α) and interleukin 6 (IL-6), which are biomarkers for aging and are secreted by both immune cells and white adipose tissue (WAT). Both levels of these inflammatory cytokines increase with age [20]. Within the WAT, the stromal vascular fraction (SVF), home to the M2-type macrophages and T-cells, secretes IL-6 [21]. This cytokine affects the metabolism, which increases the amount of the appetite-quelling hormone leptin and helps increase hepatic insulin resistance through the release of free fatty acids [19]. 

**Figure 4:**
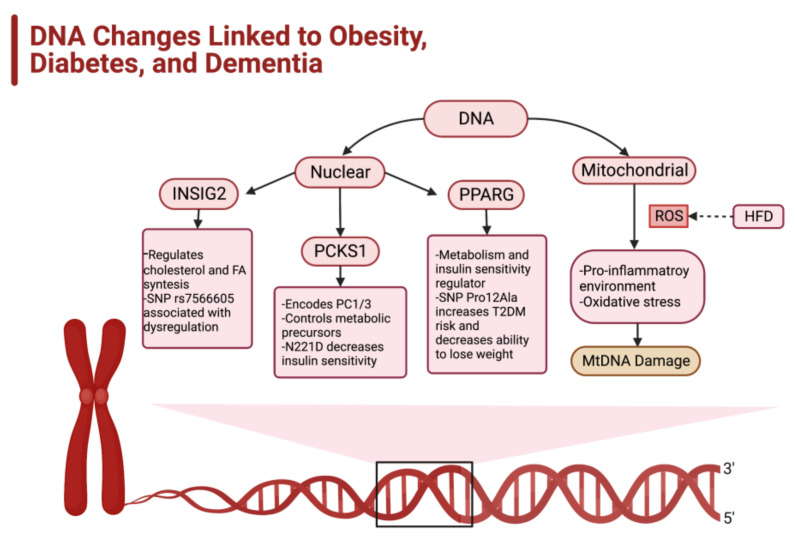
Genetic predisposition for development of obesity. The predisposition for the development of obesity is dictated by several genes, both nuclear and mitochondrial. The nuclear genes insulin-induced gene 2 (INSIG2), proprotein convertase subtilisin/kexin type 1 (PCKS1), and peroxisome proliferator-activated receptor gamma (PPARG) play a role in metabolism regulation. Mutations or single nuclear polymorphisms (SNPs) cause the dysregulation of these genes and thus awry metabolic function that can stimulate the development of obesity.

Age is believed to be correlated with two other important molecules—adiponectin and leptin. These two are thought to be impacted by the central redistribution and increase of WAT, while also playing a role in regulating metabolism (Figure 4). Adiponectin levels positively correlated with age, and in the Li et al. (2021) mice study, adiponectin proved key in determining “healthspan” and lifespan [22]. This adipokine is unique in that it has an antagonistic impact on obesity. Although it is secreted by adipocytes, adiponectin increases the rate of lipid metabolism and also has insulin-sensitizing and anti-inflammatory effects [19]. Obesity lowers levels of adiponectin, furthering the perpetual inflammatory state and insulin resistance [18].

Leptin is another adipokine that combats obesity. Leptin lowers appetite by communicating with the satiety centers of the brain and stimulates fat oxidation [23]. Obese individuals tend to have increased levels of leptin; however, while this might seem to be beneficial, it is not, because it leads to leptin resistance [24,25]. Research indicates that individual leptin levels increase with age, and although evidence shows leptin protects against cognitive decline, this only seems to hold true for those with a healthy BMI, likely due to leptin resistance [26]. Women seem to benefit more from the protective effects of high leptin levels. The development of neurodegenerative disorders, particularly AD, are associated with low leptin levels [25,26].

### 2.2. Gender and Obesity

Gender is a major factor in the development of obesity. Most women are twice as likely to become overweight or obese compared to men. Women also have a far greater chance of suffering from obesity-related complications with double the mortality rate [27]. A study that compared 1178 South Asian adults found a significantly higher rate of obesity among female participants, with women being 2.5 times more likely to develop centralized obesity compared to their male counterparts [28]. Women are disproportionately affected by obesity and obesity-related complications due to the impact of their emotional state (e.g., negative emotions correlate with stress eating), hormones, and degree of physical health. States of emotional flux or distress are associated with an increase in caloric intake in women. Research indicates these negative emotional states trigger higher incidences of food cravings in both males and females [29]. However, the reported foods craved by females are categorized as “sweet” and are typically more calorically dense (e.g., chocolate, ice cream), whereas males in the study craved more protein rich “savory” foods (i.e., meat). At the biological level, hormones offer another potential explanation for the discrepancy in obesity rates between men and women. Androgens (i.e., testosterone) tend to reduce the development of diabetes and obesity in men [30]. Estrogen, the predominate hormone in females, is linked to a reduction in appetite and inflammation in women. Postmenopausal women showed an increased propensity to gain weight following a decrease in estrogen levels [30]. The clinical determinants, including pregnancy, hormonal birth control (chronic contraceptive use is associated with a higher body weight and adiposity), menopause, and polycystic ovary syndrome (PCOS) also contribute to the variance between men and women. In addition to generally having more pre-dispositional factors, it is important to note that women also have a higher rate of mortality due to obesity-related complications because they have a higher risk of developing these complications [27]. Dementia is one such complication, and given that *JAMA* reports that two-thirds of the adults diagnosed with AD are women, obesity is a pivotal modifiable risk factor [5].

### 2.3. Genetic Factors Impacting the Development of Obesity

Humans are a compilation of genes and behaviors; therefore, it is easy to understand why genetic elements impact the likelihood of becoming obese. Despite the large genetic pool that contributes to the development of obesity, this article only examines three genes commonly associated with obesity with a strong subsequent link to diabetes: the insulin-induced gene 2 (INSIG2), proprotein convertase subtilisin/kexin type 1 (PCKS1), and peroxisome proliferator-activated receptor gamma (PPARG) genes [31,32] (Figure 4). Other genes that can predispose individuals to obesity include the fat mass and obesity-associated (FTO) gene, leptin receptor (LEPR), and melanocortin-4 receptor (MCR4) [32,33].

The INSIG2 gene, found on chromosome 2q14.2, is an endoplasmic reticulum protein that inhibits sterol response element-binding protein (SREBP) activation. This prevents the binding of SREBPs to the SCREBP cleavage-activating proteins (SCAP), which blocks SREBP movement to the Golgi and ultimately impacts the regulation of cholesterol and fatty acid synthesis. There are two control points that lead to the activation of INSIG2: (1) increased high-cholesterol levels and (2) insulin induction. The single nuclear polymorphism (SNP) rs7566605 of INSIG2 is associated with INSIG2 dysregulation and a 40% increased risk of developing obesity [34,35] (Figure 4).

Another gene, PCKS1, is located on chromosome 5q15.21 and codes for the proteolytic prohormone convertase PC1/3, and controls the cleavage and activation of metabolic regulating precursors (e.g., proopiomelanocortin, proinsulin, proglucagon, proghrelin) [29]. The prohormone convertase (PC1/3) helps regulate glucose homeostasis and appetite, which predisposes individuals with mutations in the PCSK1 gene to obesity and diabetes. Specifically, the N221D allele has a strong tie to diabetes due to its deleterious effect on insulin sensitivity and oral glucose tolerance, while increasing the circulating proinsulin (Figure 4). N221D is also correlated with an increased risk for pediatric obesity [36].

A protein-coding gene, PPARG, is a gene that, once translated, yields a nuclear ligand-dependent transcription factor that is found in adipocytes. The PPARG gene serves as a key metabolic regulator that aids in controlling adipogenesis, lipid storage and metabolism, thermogenesis, and insulin sensitivity [37]. This gene is associated with the “browning” of WAT into brown adipose tissue (BAT), a special type of adipose tissue that is credited with regulating thermogenesis due to an upregulated amount of the uncoupling protein 1 (UPC1) gene. BAT, in essence, is a type of fat that “burns” itself off to maintain thermogenic homeostasis [38]. The importance of this gene in multiple metabolic processes allows it to be detrimental in the case of certain variants, such as the SNP Pro12Ala that leads to an increased risk of developing both obesity and type 2 diabetes [37] (Figure 4). Studies indicate that individuals who possess the Ala12 allele consistently maintain a steady body weight and insulin sensitivity. In contrast, individuals who possess Pro12Ala mutated allele exhibit a greater difficulty with weight loss [39].

### 2.4. Epigenetics and Predisposition for Obesity

The genetic code lays the foundation for an increased tendency towards obesity, but the epigenetic alterations made to the genome are fascinatingly pivotal pieces of the puzzle. Epigenetics can be defined as heritable alterations that impact gene expression and function, but do not change the actual genetic code. This process occurs through methylating and acetylating the deoxyribonucleic acid (DNA) or histones or alteration of the micro-RNA (miRNA). Methylation generally compacts and silences DNA, while acetylation generally activates DNA. miRNA serves another means of lowering DNA expression by acting as a blockade and preventing any enzymatic activity [40]. Epigenetics affects every aspect of one’s health and can be altered in a variety of ways, through maternal actions during and prior to the gestational period and later through diet and exercise. Therefore, epigenetics can be a two-way street—some alterations may predispose individuals to obesity, but obesity and the lifestyle that stimulates it can also cause these genomic changes and further perpetuate obesity.

The maternal state of health heavily dictates embryonic development and the health of a newborn. However, several studies suggest that the impact of a mother’s health and lifestyle conditions can have long-lasting effects and can predispose their child to obesity. Most interestingly, this is illustrated in a small study that compared the offspring of obese mothers who underwent gastric bypass surgery [41]. The children born prior to the mother’s gastric bypass surgery had greater obesity attributes than their siblings born after the surgery and maternal weight loss. The study found differences in the methylation profiles when comparing the metabolic and insulin-regulating genes [41]. Other examinations reveal key areas of the genome responsible for a rise in adiposity. The retinoid X receptor alpha gene (RXRα), which is PPARG’s heterodimeric partner, works in the adipocyte to regulate adipogenesis and metabolic processes [37]. The methylation of RXRα at birth increases the risk of obesity, which explains about 26% of the variation in childhood obesity.

The insulin-like growth factor 2 (IGF2) also plays a role in embryonic development. This gene is classified as an “imprinted” gene from a paternally derived allele, with the maternal allele silenced. The methylation of IGF2 at H19 is associated with an increased risk of early-onset obesity in children and higher circulating levels of the protein encoded by IGF2 in adults [42]. Although this is a paternally derived allele, maternal actions dictate the status and impact of methylation. Maternal breastfeeding may blunt the effects of H19 methylation [42]. Regular maternal exercise during the gestational period may also blunt the levels of methylation found on the IGF2 gene [43]. 

Diet and nutrition are key mediators of obesity and can also incite changes in epigenetic factors. Caloric surplus is the biggest cause of obesity, but particular types of diets are more prone to inducing a greater risk of obesity. A high-fat diet (HFD), for example, is linked to altering the epigenome and raising the risk of obesity. A clinical trial utilizing an HFD for five days found an increase in the methylation of PPARG [44]. Other notable epigenomic changes occur with an increased expression of histone deacetylases (HDAC): HDAC5, HDAC8, and HDAC9. These enzymes alter the expression of the genes responsible for metabolism and appetite regulation, leading to an increased propensity for obesity [44].

### 2.5. Socioeconomic Status and Obesity

Obesity is a socioeconomic problem as well as a biological problem. Those of a lower socioeconomic status are disproportionately affected by the obesity epidemic due to a lower quality diet and lack of resources (e.g., access to workout facilities and supermarkets, proper health/nutrition education, time) [38]. Minority groups are hit the hardest by these disparities, especially within rural areas. Healthier food options tend to be more expensive than processed, calorie-dense food options. These cheaper options are also easier to access than more nutritious options due to the lack of supermarket availability in lower socioeconomic populations. The use of government assistance food stamp programs also has a correlation with a higher BMI due to the high sugar and fat content of the food offered. Along with less access to supermarkets, minority groups also have less access to physical activity centers (e.g., gyms). This works detrimentally in tandem with the decline in safe and well-maintained outdoor recreational spaces and the decrease in walking as a form of transportation [45].

## 3. Diabetes and Obesity

**Figure 5:**
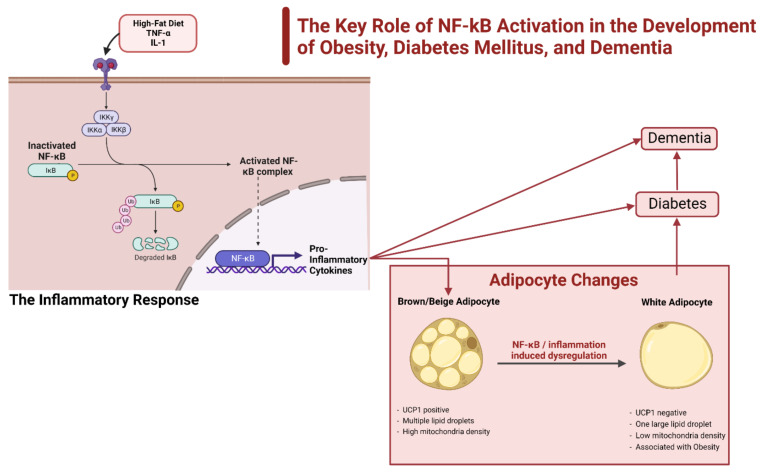
Role of NF-κB activation in the development of obesity, diabetes mellitus, and dementia. Activation of NF-κB is a nuclear transcription factor linked to the ignition of inflammation via the release of inflammatory cytokines (e.g., IL-1, IL-1β) that leads to the dysregulation of several pathways throughout the body. One noteworthy impact of this inflammation is the transformation of brown adipose tissue (BAT) to white adipose tissue (WAT). High WAT content is a hallmark of obesity, and from obesity, diabetes and dementia develop. Note that inflammation lends itself to the direct development of both diabetes and dementia.

The previously discussed factors all perpetuate obesity and thus diabetes; however, inflammation is the key factor and mediator of both obesity and diabetes. Although a state of caloric surplus promotes an inflammatory state, studies indicate that the immune system plays a role as well through the following: (1) central nervous system (CNS) inflammation, (2) TLR activation, and (3) certain gut microbiota [46]. This inflammatory response mediates various components of the immune system, most notably macrophages and T-cells. There are currently three working hypotheses on how adipocytes are stimulated within the immune system: (1) adipocytes secrete cytokines that recruit monocytes (become M1 macrophages) and T-cells, (2) an HFD stimulates the toll-like receptor 4 (TLR4) and mounts an immune response, and (3) adipocyte death recruits macrophages to clean up necrotic cells [46,47]. This section specifically discusses the role of NF-kB and related pathways, CNS control and the impact of inflammation, and the types of diabetes and their subsequent relationship to inflammation and dementia (Figure 5).

### 3.1. NF-κβ and the Impact of the Inflammatory Response

NF-κB is a transcription factor that enters the nucleus and acts as an inflammatory mediator by upregulating various pro-inflammatory genes. The dysregulation of this gene leads to the runaway inflammation found in several pathological conditions (Figure 5). NF-κB is modulated in one of two ways: the canonical and non-canonical pathway. The canonical pathway is handled by the IKK complex composed of IKKα and IKKβ, and NF-κB essential modulator (NEMO) or IKKγ [48]. The IKK complex can be stimulated by extracellular stimuli, but most predominantly by TNF-α and IL-1 [42]. After activation, IKK phosphorylates the molecule IκB, an inhibitor bound to NF-κB, and releases NF-κB to regulate gene expression within the nucleus [46,48] (Figure 5). The non-canonical or alternative pathway begins with the activation of NF-κB-inducing kinase (NIK). NIK then phosphorylates IKKα, which stimulates the processing of the p100 protein into p52, the active subunit that then binds RelB. Following activation, RelB enters the nucleus and upregulates its target genes [49,50].

The non-canonical pathway is the pathway that is highly associated with obesity and diabetes. A 2009 study designed to increase the obesity of obese mice on a high-fat diet identified another subunit of IKK called IKKε. Mice with the IKKε gene removed that were fed a high-fat diet that induced obesity showed an upregulation of UPC1 in BAT and had normal insulin sensitivity [51]. The results of this study link IKKε directly to an induction of obesity on a high-fat diet and indicate that the lack of IKKε has protective effects. The IKKε gene is induced by a caloric surplus and transcriptionally by NF-κB. Type 2 diabetes also increases in the activation of NF-κB due to the chronic inflammation already present in the other immune cells and adipokines, hyperglycemia, TLR4 activation by the high circulation of free fatty acids, and stress/damage to the endoplasmic reticulum [50] (Figure 5). A possible explanation for the increased activation of the non-canonical NF-κB is an increase in the activating ligands CD40L, LIGHT, and lymphotoxin found in diabetic patients. Increased activation of the non-canonical pathway appears to be detrimental to the insulin-producing β-cells [50]. One study examined another aspect of the non-canonical pathway believed to contribute to the development of diabetes from the chronic inflammatory state of obesity. The NIK was overexpressed in the hepatocytes of obese mice, leading to hyperglycemia due to increased glucagon activity and an environment of glucose intolerance [52].

### 3.2. CNS Control and the Impact of Inflammation

Research indicates that a high-fat diet is a risk factor for diabetes in many ways, which has a far-reaching impact within the CNS, particularly the hypothalamic region of the brain. The hypothalamus regulates metabolic homeostasis and determines energy intake stimulation (e.g., hunger) through hormones like leptin and ghrelin [53]. Inflammation within the CNS progresses rapidly and begins days after initiating a high-fat diet. This inflammation first impacts the hypothalamic arcuate nucleus (HAN) and causes injurious effects to metabolic homeostasis in the form of dysregulating leptin levels which ignites insulin resistance. Benomar identified that an increase of TLR4 in obese mice caused dysregulation due to a rise in the available saturated fatty acids [53]. The TLR4 activation impacts the proopiomelanocortin (POMC) neurons located in the HAN through microglial action. The POMC has a role in the anorexigenic effect on the regulation of energy expenditure and food intake via hormones such as leptin [54,55]. Leptin levels dictate the expression of POMC, with increased levels of leptin leading to increased expression of POMC, decreased appetite, and increased metabolic rate. Another key population of neurons within the HAN is the orexigenic (AgRP) that upregulates appetite when stimulated, partially through the actions of ghrelin. Ghrelin is one of the stimulators of hunger and AgRP neurons. The AgRP neurons sense hunger, increase adipose tissue, and reduce metabolic rate [56,57]. The inflammatory actions of microglia activated by TLR4 impact these AgRP neurons. Microglia action in the AgRP neuronal population has an inhibitory effect, which suppresses the effect. This contradictory effect serves as a short-term protective mechanism against the inflammation in the HAN caused by a high-fat diet. However, chronic inflammation appears to cause microglia to contribute to the damage [55,58].

### 3.3. Role of Inflammation in Diabetes and Dementia

Alzheimer’s disease (AD), the result of “type 3” or “brain” diabetes, displays a characteristic cognitive decline due to the amyloid fibril plaques and neuronal necrosis [13,14,15,16,59] (Figure 3). Obesity and diabetes are significant risk factors for developing the disease because a key mediator of the disease progression is insulin resistance within the brain that contributes to the creation of the neurofibril tangles and necrosis. A misfolded version of the brain-specific protein, tau, creates these tangles. Tau regulates insulin and insulin growth factor-1 (IGF1); resistance to these two in the brain causes the upregulation of glycogen synthase kinase 3β (GSK-3β). The increased activity of the kinase GSK-3β hyper-phosphorylates tau and leads to misfolding and neurofibril aggregates [60]. The hippocampus, associated with memory, is one of the areas with the highest concentrations of the insulin receptor (IR), which links diabetes to AD [16]. Oxidative stress, an imbalance between the reactive oxygen species and the system’s ability to negate these free radicals, can propagate the progression of disease in diabetes and AD. Increased oxidative stress leads to apoptosis due to mitochondrial dysfunction, peroxisomes, and damage to the cellular membrane. This cell death contributes to the cognitive decline seen in patients with AD [16,61].

## 4. Current Status of Research on Obesity, Diabetes, and Dementia

Research in the areas of obesity, diabetes, and dementia is ongoing and continues to progress. Although the understanding of these conditions has grown rapidly in recent years, much is still unknown, which leaves researchers with a dual goal of increasing the knowledge of each disease as well as devising new treatments. Medications targeting the POMC-MCR pathway, the traditional diabetes drug metformin, and the molecule Pdia4 are all current research interests.

### 4.1. Studies on the POMC-MCR Pathway

The POMC neurons and associated leptin–melanocortin pathway is currently a mainstay of obesity-based genetic research. Scientists believe the effect of the POMC gene offers protection against the development of T2DM [62,63]. The POMC gene is a precursor for three melanocortin peptides known as melanocyte-stimulating hormones (MSH)—α-MSH, β-MSH, and γ2-MSH—and the molecule β-endorphin. These peptides and the molecule interact with melanocortin receptors (MCRs) to decrease appetite and increase the metabolic rate [62]. Recent rat model studies indicate that rats deficient in the POMC gene develop obesity, glucose intolerance, and insulin resistance [58]. Older rat model studies clearly identify α-MSH to be a potent appetite suppressor and stimulator of TRH through the activation of MC4R [63]. In 2006, Tung et al. injected POMC-null mice with either α-MSH, β-MSH, or γ2-MSH and allowed the mice to free feed. Mice treated with α-MSH showed the largest decrease in body mass and food intake after a single dose compared to mice treated with β-MSH and γ2-MSH [62,64]. Because rodents do not express β-MSH due to a lack of an N-terminal proteolytic site that allows cleavage and production of the protein, the β-MSH effects were unclear.

More recent studies elucidated the role of β-MSH via the Labrador retriever animal model [60]. Compared to the rat model (69%), the canine model (79%) offered a greater degree of genetic similarity in the POMC gene because Labrador retrievers have the highest prevalence of obesity among dog breeds and have similar comorbidities as humans. In 2016, Raffan et al. studied a cohort of 310 Labrador retrievers classified as assistance dogs to determine the effect of the naturally occurring 14-bp deletion of the POMC gene. This deletion prevents the production of β- on POMC-MCR MSH and β-endorphin, both of which are associated with hyperphagia, increased adiposity, and loss of appetite control. Labradors with the mutation showed higher body weight and increased food intake motivation behaviors [65].

#### Drugs Utilizing the POMC-MCR Pathway for Obesity Treatment

Scientists are investigating new obesity drugs based on the POMC-MCR pathway studies to further illuminate the genetic mechanisms partially responsible for the development of obesity [66]. MC4R agonists, designed to stimulate the MC4R in place of the POMC ligands, are a well-researched therapeutic route that reduces adiposity and appetite; however, most of these agonists have cardiovascular drawbacks, such as hypertension. Two MC4R agonists under research are LY2112688 and a new FDA-approved drug called Setmelanotide (BIM-22493).

In a double-blind pharmaceutical trial, researchers administered the MC4R agonist LY2112688 to a mix of obese men and women who were either MC4R deficient or MC4R sufficient (control group). Administration of LY2112688 yielded a higher incidence of hypertension in the MC4R-deficient group than the control group [66]. However, not all MC4R agonists have a hypertensive effect [67]. The new drug, Setmelanotide, is an eight-amino acid cyclic peptide labeled as BIM-22493 or RM-493 that functions as an MC4R agonist to treat the monogenic causes of obesity caused by POMC deficiency by lowering food intake and restoring insulin sensitivity [67,68].

A phase II, non-randomized, open-label human study conducted in 2016 by Kühnen et al. examined the effects of Setmelanotide in two obese POMC-deficient patients. Patient 1 was a 21-year-old female with a heterozygous loss-of-function mutation in the POMC gene and early-onset childhood obesity. Patient 2 was a 26-year-old female with a homozygous loss-of-function mutation in the POMC gene. Both patients reported a 9/10 score on the Likert hunger scale (0 = no hunger, 10 = extreme hunger), indicating a high degree of hyperphagia. Both patients were injected subcutaneously once daily with Setmelanotide. Patient 1 received 0.25 mg and Patient 2 received 0.5 mg, with both patients receiving incremental dose increases of 0.5 mg per week. Both patients reported a decreased appetite with increased dosages, with their Likert hunger scores reported as a 0 or 1 at a dose of 1.5 mg. Both patients exhibited significant weight reduction while on the drug. However, the cessation of Setmelanotide resulted in an increase in Patient 1’s hunger levels to a 7 on the Likert hunger score and a weight gain of 4.8 kg. Minimal side effects (e.g., dry mouth, pain at injection site) were reported and did not include any hypertensive effects [67,68].

Tests of Setmelanotide on Rhesus macaques resulted in a 35% transient decrease in intake and a 13.5% body mass weight loss in the 8-week treatment course. The results indicated that 0.5 mg/kg/day dosing of BIM-22493 in male Rhesus macaques reduced food intake. However, this effect was only temporary after the treatment stopped. A comparison of LY2112688 and BIM-22493 in primates revealed that a comparable dose of BIM-22493 had a higher decrease in food intake than LY2112688 [69]. Notably, BIM-22493 did not alter either blood pressure or heart rate. Similar studies conducted with mice, rats, and dogs yielded comparable weight loss results [69,70].

### 4.2. Effect of Metformin in the Treatment and Prevention of Dementia

Studies on metformin, the drug of choice for T2DM, show promising improvements in cognition as well as disease progression [71]. Metformin, derived from the plant *Galega officinalis* (specifically the product galegine), has a mechanism of action that is not fully understood. Researchers believe metformin either inhibits mitochondrial respiration, mitochondrial glycerophosphate dehydrogenase, or alters lysosome activity likely using an AMP-activated protein kinase (AMPK)-dependent mechanism [72,73]. Due to the close link between T2DM and AD, a double-blind, randomized, placebo-controlled crossover pilot study examined the effects of metformin on the CNS in non-diabetic AD patients with mild cognitive impairments. The data collected indicated that metformin penetrates the blood–brain barrier (BBB) as measured in the CSF. Metformin-treated patients showed improved cognition related to memory, learning ability, and attention/focus abilities. The placebo group did not yield these same results [74].

A more recent 2021 study investigated the cognitive and physiological effects of metformin in late middle-aged mice [73]. The 10-week trial used 59 male C57BL6/J mice. The treatment group received oral 100 mg/kg/day doses of metformin. Mice treated with metformin displayed improved memory and functioning, general and neural anti-inflammatory effects, and increased AMPK activation [73]. The improved memory seems to be linked to the increase of autophagy within the hippocampus, as lower rates of autophagy are associated with aging and impaired cognitive function. Neuro-inflammation is another hallmark of aging, particularly within the hippocampus [75,76]. This inflammation is mediated by the immune cells within the brain, microglia, and astrocytes that release pro-inflammatory cytokines. The examination of neural tissue post-metformin treatment revealed microglia activated to the anti-inflammatory M2 phenotype and reduced amounts of microglia present, which led to a lower measured concentration of the cytokines TNF-α and IL-1β [75]. Another pertinent anti-inflammatory effect was metformin’s inhibition of NF-κB in the treated mice [73].

### 4.3. Studies Utilizing Pdia4 to Treat Diabetes

Recent research offers novel insight into the understanding of the pathogenesis of diabetes and could yield a new treatment. A 2021 study utilized a mouse model to explore the role of protein disulfide isomerase family A, member 4 (Pdia4), in the regulation and dysfunction of β-cells and the role it plays in the development of diabetes. Pdai4 is a member of the protein disulfide isomerase (PDI) family [77]. This family of proteins serves as oxidoreductases and chaperones that catalyze disulfide bonds, maintaining other proteins conformations, and regulating other protein interactions. Malfunctions in PDIs lead to various diseases, such as diabetes, obesity, dementia, cancer, and cardiovascular pathologies [78].

The Kuo et al. (2021) study revealed that Pdia4 was paramount in ROS production, leading to pancreatic β-cell failure and the development of diabetes [77]. The study also showed that the use of Pdia4′s inhibitor, 2-β-D-glucopyranosyloxy1-hydroxytrideca 5,7,9,11-tetrayne (GHTT), prevented the activation of the pathway responsible for ROS production and improved diabetic symptoms via improvements in β-cell function. Scientists made these conclusions using a detailed mouse model utilizing *Lepr db/db* mice, which model phase 1 to 3 of T2DM and obesity. The *Lepr db/db* mice had an increased expression of Pdia4 in their pancreatic β-cells. Following this discovery, the researchers bred mice with the Pdia4 gene knocked out, with Lepr db/db mice and their offspring, which were Pdia 4-/-Lepr db/db, exhibiting borderline diabetes with a later onset than *Lepr db/db* offspring with the Pdia4 gene present. The researchers gave the Pdia4 inhibitor, GHTT, orally to *Lepr db/db* mice in 1-, 5-, and 25-mg/kg doses from 8 to 24 weeks of age. GHTT reduced the pancreatic β-cell atrophy and ROS production induced by Pdia4 [77]. The role of Pdia4 and its inhibition in the pathogenesis and potential slowing of diabetic progression is promising and warrants further research.

## 5. Diets That Affect Obesity

**Figure 6:**
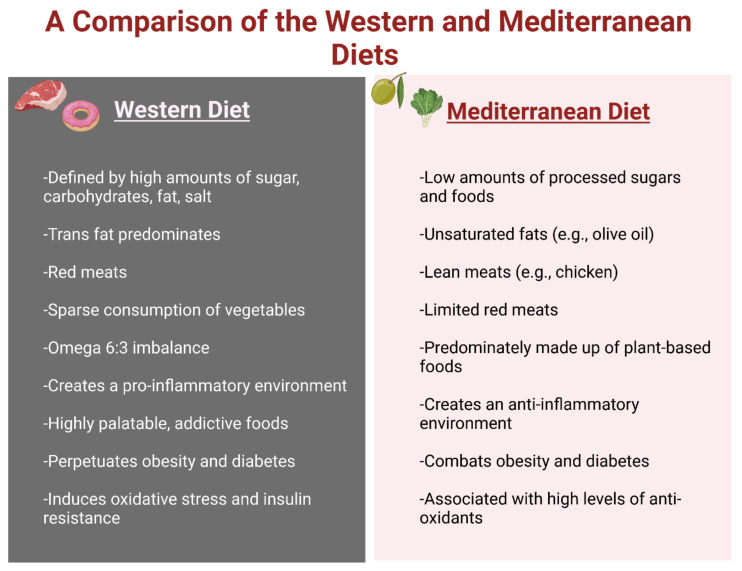
Comparison of the Western diet versus the Mediterranean diet. The Western diet and its associated fatty foods are heavily linked to the causation of obesity and insulin resistance due to the induction of oxidative stress through the creation of reactive oxidative species and the pro-inflammatory environment that it creates. In contrast, the Mediterranean diet is low in fat, sugar, and red meat, and offers plant-based options. This leads to a greater concentration of antioxidants, which combat ROS and soothe inflammation.

Obesity is derived through a combination of genetic and environmental factors. Although genetics can predispose individuals to obesity, the evolution of nutrition (particularly the Western diet) is a major contributor to the issue (Figure 6). As discussed, obesity is an inflammatory condition. Certain diets are pro-inflammatory, which promote the development of obesity, diabetes, and thus dementia [79,80]. These pro-inflammatory diets cause an increase in reactive oxygen species (ROS), insulin resistance (IR), and dysregulation in the activation of the sympathetic nervous system (SNS) and the renin–angiotensin–aldosterone system (RAAS) that contributes to the increase in obesity and related conditions [81]. The Western diet (WD) is the most prominent of these pro-inflammatory diets; however, diets such as the Mediterranean diet (MD) lower inflammation and decrease health risks.

### 5.1. Western Diet (WD)

The ideal diet to maintain a healthy weight as recommended by the CDC’s “Dietary Guidelines for Americans 2020–2025” includes the following: 2.5 cups of vegetables, 2 cups of fruit, 6 ounces of grains, 3 cups of dairy, and 5.5 ounces of protein a day (avoiding red meat) [82]. These guidelines also stipulate that caloric intake should be 240 kcal or lower a day, with less than 10% of the caloric intake from either added sugars or saturated fats [82]. Furthermore, the American Heart Association recommends less than 6 teaspoons of sugar per day for men and less than 9 teaspoons for women [83]. It is also important to note that these guidelines can be adjusted for age, gender, level of physical activity, and other altering factors. Although these guidelines offer a balanced way of eating, this is not the prominent diet within the US or the Western world. Instead, the dominant WD correlates with the rise in obesity [82]. The WD is the antithesis of the recommended dietary guidelines. The bulk of the caloric intake in the WD comes from food rich in sugar, saturated and trans fats, carbohydrates, salt, and red meat [82]. The WD also deemphasizes vegetables and fruits. One of the major contributors to the pro-inflammatory environment is the imbalance of omega-6 to omega-3 ratio. A high ratio, caused by a dramatic increase in omega-6 levels, is pro-inflammatory (Figure 6). The emphasis on saturated and trans fats and vegetable oils in the WD diet creates an average ratio of 20:1 [81].

Research indicates the continued implementation of the WD, which is based on desirable, high-calorie foods and causes addictive behaviors such as binge or stress eating through disturbances in hypothalamic–pituitary–adrenal axis regulation [84,85]. This disturbance starts the cycle that creates obesity and related cognitive problems. The hippocampus handles memory, but it also plays a role in decision making and the WD-induced disturbances lower the ability of the hippocampus to regulate food intake, leading to overeating [84,86]. This hippocampal disturbance and the addictive behaviors stimulated by the high-fat, sugary foods are a powerful combination that perpetuates obesity [84].

Oxidative stress and insulin resistance are two major problems prompted by the WD [87,88]. The high-sugar, calorically dense foods prompt more insulin release, but the timing of food intake is also important. Often the WD includes constant snacking, leaving the body in the postprandial state, with constantly elevated insulin levels. This hyperinsulinemia is a likely cause of IR, and thus contributes to the development of T2DM and obesity [88,89]. The constant postprandial phase also contributes to the creation and imbalance of ROS free radicals, which creates a pro-inflammatory environment and also helps derive IR. The nutrient excess of the WD increases ROS [87]. Excessive nutrients translate into an increased electron supply, which, without an increase in demand for energy molecules (ATP), leads to a raised mitochondrial membrane potential. This increase in membrane potential causes the increase in ROS during oxidative phosphorylation [81]. This mechanism is a contributor to the NF-κB-mediated inflammation and IL-6 release from adipocytes [90].

The activation of the SNS and RAAS are also contributors to IR and the inflammatory state. The SNS serves as a regulating system using the catecholamines epinephrine and norepinephrine to control several physiological aspects, such as the resting metabolic rate and catabolic metabolism, thermogenesis, food intake, and blood pressure [91,92]. Obese individuals have increased SNS activity, which translates into hypertension and increased insulin resistance [93]. Insulin serves as an activator of the SNS and hyperinsulinemia causes a radical increase in SNS activity that leads to catabolism, hypertension, and an increase in RAAS activity. The RAAS system is a hormonal regulatory system based in the kidneys and helps to maintain blood pressure. These system effects are mediated by the peptide angiotensin II (ANG II), which is the product made from the breakdown of angiotensinogen. The RAAS system may have a basis in the kidneys, but it is important to note that adipocytes can make up to 30% of the body’s angiotensinogen and begin the RAAS cascade [94]. The overactivation of the RAAS system perpetuates hypertension and IR through the induction of oxidative stress, which cycles back around to the inflammatory state associated with obesity, T2DM, and AD [81].

### 5.2. Mediterranean Diet (MD)

In contrast to the WD, the MD is anti-inflammatory and has several health benefits, with research indicating that it is the best diet to combat obesity [95,96]. The MD is defined by the consumption of a majority of plant-based foods (e.g., vegetables, fruits, nuts, seeds, olive oil), low to moderate amounts of dairy and lean meat (e.g., poultry, fish), and minimal amounts of red meat and eggs [96]. The MD has a positive correlation with antioxidants (e.g., glutathione peroxidase, superoxide dismutase) that offer protection against the generation of ROS [97] (Figure 6). The health benefits, if followed during pregnancy, include the decreased risk of neural tube defects in the fetus and a reduced tendency for the offspring to become obese, likely through epigenetic mechanisms [96]. Studies indicate that the MD is effective in inducing weight loss, particularly when dieters switch from the WD to the MD. However, a 2020 study found that participants who followed the MD were twice as likely to maintain their weight loss [98].

## 6. Current Therapeutics

Obesity, diabetes, and dementia are all interrelated, so there is no surprise that their treatments are also interconnected in various ways [99]. Obesity is an imbalance of energy input and energy expenditure at the most fundamental level. The current baseline treatment plan, assuming no other underlying conditions exist, is to correct this imbalance with a caloric deficit. A caloric deficit can be created by a combination of lowering caloric intake and increasing physical activity. Although a caloric deficit is paramount, nutrition alterations are also recommended [96]. Healthier food choices, such as switching from the WD to the MD, or a more plant-based diet, offer more benefits that simply losing weight (Figure 6). A healthier diet lowers the risk of developing cardiovascular conditions and dementia [96]. Current guidelines recommend losing 5–10% of body weight within the first 6 months [100]. The combination of calorie restriction and incorporating antioxidant-rich foods helps decrease the inflammatory state and causative oxidative stress [100].

### 6.1. Diet and Lifestyle Modifications

The implementation of lifestyle modifications is paramount to prevent disease. Obesity causes chronic health conditions that can drastically shorten and lower the quality of life [1,2]. All of these chronic conditions could be managed or prevented by following a more plant-based diet and introducing regular exercise, especially if implemented at a young age [1]. The current increase in the use of the WD has led to a rise in obesity, especially in children and expectant mothers. Within the United States, the obesity prevalence among children aged 2–19 years was 19.7% during the years of 2017–2020 and this percentage has continued to steadily increase [101]. Obesity at a young age is extremely detrimental as children usually become obese adults with early development of diabetes and all of its complications, including dementia [102]. The prevention of obesity using proper nutrition management and physical exercise should begin at a young age to prevent complications. These small changes in childhood can manifest good results: (1) avoid overfeeding infants (bottle feeding is associated with a greater risk of excessive caloric intake), (2) reduce sedentary and electronic use time, (3) aim for 1 h of physical activity per day for children 3–5 years of age, and (4) limit children’s fat intake to less than 30% of caloric intake [103]. In adulthood, obesity lifestyle modifications remain the gold standard for treatment. Calorie restriction is the foundation for the treatment and prevention of obesity, diabetes, and dementia [104].

Additional dietary interventions (e.g., certain vitamins and nutrients frequently found in plant-based foods) in tandem with calorie restriction is especially promising at subverting AD [102]. Dietary adjustments, such as incorporating foods rich in omega-3 polyunsaturated fatty acids, curcumin, flavonoids (natural components found in plants), minerals (e.g., calcium, magnesium, potassium), and drinking caffeine are proven to lower the risk for AD by increasing the antioxidants in the body and lowering the amount of inflammation within the body and brain [102,105,106,107,108,109]. Although the incorporation of foods rich in anti-oxidants is important and offers several anti-aging health benefits, cutting out high-fat, processed, and sugary foods is also critical.

Another key lifestyle modification is the incorporation of or increase in physical activity. Regular exercise offers several health advantages, including a reduction in body weight, better glycemic control, a reduction in dyslipidemia, and a decrease in the amount of Aβ aggregates that are associated with cognitive decline and AD [102,110,111]. Exercise can take many forms, such as 30-min daily walks, weight training, or playing sports with friends [112]. The utilization of consistent lifestyle changes offers society the chance to prevent chronic conditions such as obesity, diabetes, and dementia, and also serves as the primary method of treatment.

### 6.2. Pharmaceutical and Surgical Options for Obesity Treatment

**Figure 7:**
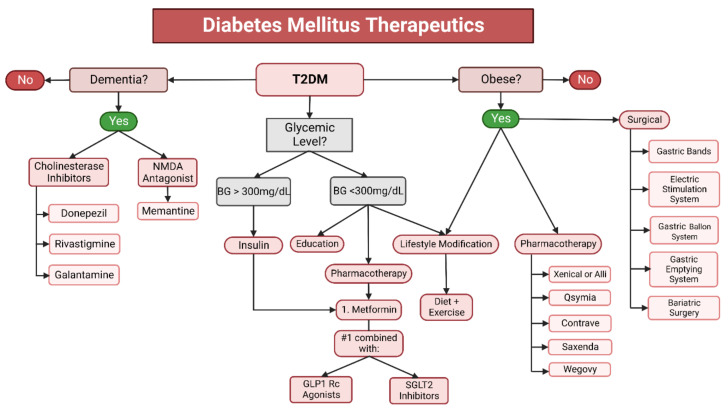
**Similarities of the therapeutics for obesity, diabetes mellitus, and dementia**. Because obesity, diabetes, and dementia are so closely related, several aspects of their treatments overlap. Lifestyle modifications (e.g., change in diet, increase in exercise) are the most effective means of treating or preventing all three conditions; however, several medications and surgical options are available to aid in the management and treatment of these conditions.

There are also a variety of other treatment options to aid the weight loss process, such as medications, weight loss devices, and bariatric surgery. These additional methods are meant to be used in conjunction with a change in diet and increase in activity, and not to replace traditional weight-loss methods. As of 2021, the Food and Drug Administration (FDA) has approved five medications for this purpose: orlistat (Xenical, Alli), phentermine/topiramate (Qsymia), naltrexone/bupropion (Contrave), liraglutide (Saxenda), and semaglutide (Wegovy) [113]. The drugs have the following mechanism of actions: (1) Xenical and Alli remain active in the gut and lower fat absorption, (2) Qsymia suppresses appetite by stimulating the sensation of satiety, (3) Contrave aids in addiction cessation and lowers appetite, (4) Saxenda lowers appetite by mimicking the hormone glucagon-like peptide-1 (GLP1), which acts in the brain to regulate food intake, and (5) Wegovy, which also controls the appetite via GLP1. These medications are currently approved for long-term use [113,114]. The FDA has also approved four weight loss devices: (1) gastric bands placed around the upper portion of the stomach, (2) electrical stimulation systems placed in the abdomen that block gastric and CNS communication, (3) gastric balloon systems (an inflatable balloon placed into the stomach and once inflated it both lowers the area available in the stomach and delays gastric emptying); and (4) gastric emptying systems involving the insertion of a tube into the stomach that drains food out of the abdomen [114] (Figure 7).

Weight-management devices are another method of regulating body weight, with two currently FDA-approved: (1) an oral removable palatal space-occupying device that limits food intake by forcing smaller bite sizes and slowing eating, and (2) ingested, transient space-occupying devices that temporarily enter and fill the stomach [114]. In the cases of severe obesity (BMI > 40 or BMI of 35–40 with comorbidities), bariatric surgery is a potential therapeutic option. The bariatric surgical procedure decreases the stomach size and alters how food moves through the gastrointestinal system. This type of surgery is generally done by either the Roux-en-Y gastrojejunostomy or sleeve gastrectomy method. Although this procedure does show excellent weight-loss results, it is not without risk [115,116].

### 6.3. Therapeutics for Diabetes Mellitus

The management of diabetes is dependent on whether it is classified as type 1 or type 2, with weight loss recommended for both types if the patient is classified as overweight or obese. In accordance with the American Diabetic Association 2022 guidelines, insulin administration is the primary treatment for type 1 diabetes [117]. The regimen is currently stated as a basal amount of insulin with mealtime and correctional insulin. It is recommended to use a long-acting insulin analog for basal insulin management because it is associated with steadier plasma concentrations and fewer hypoglycemic effects [117]. Recently, the FDA approved two hybrid closed-loop pump systems with evidence that this automated insulin injection system puts patients in their target glycemic range more efficiently and for a longer period of time compared to the alternative senor-augmented pump. Non-insulin-based treatments for type 1 diabetes include glucose-lowering drugs (e.g., Pramlintide, GLP1 receptor antagonists, sodium–glucose cotransporter-2 (SGLT2) inhibitors) and pancreas and islet transplantation [117].

T2DM is patient-dependent and managed through pharmacological methods and lifestyle modifications. Metformin is an oral medication that is the first-line therapy (Figure 7). It can be given as either a twice-daily immediate-release dose or as a once-daily extended-release dose. For patients with a blood glucose of over 300 mg/dL, insulin therapy can be initiated to quell the hyperglycemia, but it is preferable to use non-insulin therapies whenever possible. T2DM is a chronic disease, and over time, metformin alone may not offer sufficient glycemic control. In the event of the waning effectivity of monotherapy, other medications (e.g., GLP1 receptor agonists, SLGT2 inhibitors) can be added as needed on a patient-by-patient basis [117]. There is now evidence from the Vildagliptin efficacy in combination with metformin for the early treatment of type 2 diabetes that an early combination therapy of metformin and dipeptidyl peptidase could offer potential benefits [118]. Lifestyle modifications fall in line with the recommendations and guidelines surrounding obesity management, emphasizing healthier meals. The important distinction between merely trying to lose weight and controlling diabetes through dietary management is the removal of sugar-containing diets and lowering carbohydrate intake [119].

### 6.4. Therapeutics for Dementia

Dementia—like diabetes—is a chronic, progressive mental illness that cannot be cured; however, it can be managed with the treatment goal of maintaining and improving the quality of life. The treatment plan for dementia consists of a healthy lifestyle (regular exercise and heathy diet), pharmacological intervention, and occupational therapy. Previously, there were only two types of pharmacological treatment options available: cholinesterase inhibitors (e.g., donepezil, rivastigmine, galantamine) that raise the levels of acetylcholine and a N-methyl-D-aspartate receptor antagonist (memantine), and a glutamate regulator (Figure 7). Both drugs work to protect the brain’s ability to maintain learning activity and memories [120,121,122].

In 2021, a novel drug, Aducanumab, received FDA approval to treat AD. Aducanumab is a human IgG1 anti-Aβ monoclonal antibody that selects for Aβ aggregates, which are the basis of the disease’s amyloid plaques and neurofibrillary tangles. Aβ aggregates are thought to begin building up to two decades before the onset of symptoms. The drug is given intravenously once a month. Aducanumab is the first disease-modifying AD that can remove and prevent the buildup of amyloid plaques. The treatment offers a potential breakthrough in AD management and prevention; however, there is much current debate surrounding the drug’s use and effectivity [121]. The controversy surrounding the novel drug and associated accelerated FDA approval are founded on the uncertainty of the exact mechanism of AD. Aducanumab targets the accumulation of Aβ aggregates that are involved in the disease process, but it is unknown whether amyloid buildup is the exact cause of impaired cognition for both early-onset and late-onset AD. Furthermore, human clinical trials did not prove that the drug effectively improves memory or cognitive functioning. Human clinical trials are still ongoing to continue to explore the drug and clinical benefit in accordance with the accelerated approval track implemented by the FDA [123]. Although pharmacological therapeutics often take center stage in AD, occupational therapy is another step in general dementia management. Therapists can work with patients to help them make the environment safer (e.g., remove fall risks, sharp items), manage the behavioral changes associated with altered cognitive functioning, and help patients break down tasks into more manageable steps [120].

## 7. Conclusions and Future Directions

Obesity, diabetes, and dementia are highly prevalent chronic diseases within the US population, and the incidence of these diseases has increased over the past several decades. The dominant explanation for this trend incorporates a wide spectrum of factors, such as age, gender, genetics and epigenetics, socioeconomic status, inflammation, and diet. A search of the current literature indicates a strong positive correlation between obesity and the development of diabetes, particularly type 2. The chronic inflammatory damage caused in the brain by type 2 diabetes is so closely associated with the development of dementia that it has been labeled as “type 3” or “brain diabetes.” One of the predominant causes for the rise in these conditions is the HFD, including the Western diet (WD), which stimulates the inflammatory response mediated by NF-κB and its associated cytokines and the oxidative stress and an increase in the production of reactive oxidative species. Although lifestyle modifications (e.g., healthy diet, regular exercise) are the recommended treatment for obesity and the prevention of diabetes and dementia, other treatment options (e.g., surgery) and aids (e.g., pharmacological appetite suppressants and mechanical devices to limit food intake) are now available for prevention. 

This article discusses the role that obesity plays in the development of diabetes and dementia and presents current research on new molecular biology treatments designed to improve the treatment of obesity, diabetes, and dementia. These new treatments include: (1) Setmelanotide—an MC4R agonist used to treat genetically-stimulated obesity and (2) Pdia4 inhibitor (GHTT)—a drug used to improve pancreatic β-cell function in diabetic patients. As technology and understanding continue to develop, further research could clarify the pathogenesis of disease and offer novel treatment options.

## Figures and Tables

**Table 1:** Rising incidence of obesity and diabetes mellitus.

Year Range	Obesity Prevalence Percentage	Diabetes Mellitus Prevalence Percentage
1988–1994	20.2	8.8
1999–2002	27.6	10.8
2011–2014	34.25	12.9
2015–2018	40.45	14.4

## Abbreviations

WD	Western diet
MD	Mediterranean diet
T2DM	Type 2 diabetes mellitus
T3DM	Type 3 diabetes mellitus
CDC	Centers for Disease Control and Prevention
BMI	Body mass index
AD	Alzheimer’s disease
FTD	Frontotemporal dementia
LBD	Lewy body dementia
VD	Vascular dementia
WAT	White adipose tissue
TNF-α	Tumor necrosis factor alpha
IL-6	Interleukin 6
SVF	Stromal vascular fraction
SREBP	Sterol regulatory element-binding protein
FTO	Fat mass and obesity-related
LEPR	Leptin receptor
MCR4	Melanocontin 4 receptor
SNP	Single nucleotide polymorphism
PCOS	Polycystic ovary syndrome
INSIG2	Insulin-induced gene 2
PCKS1	Proprotein convertase subtilisin/kexin type 1
PPARG	Peroxisome proliferator-activated receptor gamma
BAT	Brown adipose tissue
UPC1	Uncoupling protein 1
miRNA	Micro-RNA
HFD	High-fat diet
IGF	Insulin growth factor
RXRα	Retinoid X receptor alpha gene
CNS	Central nervous system
HDAC	Histone deacetylase
IGF2	Insulin-like growth factor 2
TLR4	Toll-like receptor 4
NEMO	NF-κB essential modulator
GSK-3β	Glycogen synthase kinase 3β
HAN	Hypothalamic arcuate nucleus
KCNQ1	Potassium channel voltage gated KQT-like subfamily member 1 (KCNQ1)
KLF14	Kruppel-like factor 14
MSH	Melanocyte-stimulating hormone
MCRs	Melanocortin receptors
POMC	Proopio melanocortin
DNA	Deoxyribonucleic acid
ROS	Reactive oxygen species
IR	Insulin resistance
SNS	Sympathetic nervous system
GHTT	Glucopyranosyloxy1-hydroxytrideca
CRP	C-reactive protein
PDI	Protein disulfide isomerase
ER	Endoplasmic reticulum
MDD	Major depressive disorder
CFS	Cerebrospinal fluid
FDA	Federal Drug Administration
ARC	Arcuate nucleus
MRI	Magnetic resonance imaging
BBB	Blood–brain barrier
PUFA	Polyunsaturated fatty acids
AMPK	AMP activated protein kinase
US	United States
